# Mitochondrion as a Target of Astaxanthin Therapy in Heart Failure

**DOI:** 10.3390/ijms22157964

**Published:** 2021-07-26

**Authors:** Olga Krestinina, Yulia Baburina, Roman Krestinin

**Affiliations:** Institute of Theoretical and Experimental Biophysics, Russian Academy of Sciences, 142290 Pushchino, Moscow Region, Russia; byul@rambler.ru (Y.B.); rkrestinin@bk.ru (R.K.)

**Keywords:** astaxanthin, oxidative stress, heart failure, mitochondria, mitochondrial permeability transition pore (mPTP)

## Abstract

Mitochondria are considered to be important organelles in the cell and play a key role in the physiological function of the heart, as well as in the pathogenesis and development of various heart diseases. Under certain pathological conditions, such as cardiovascular diseases, stroke, traumatic brain injury, neurodegenerative diseases, muscular dystrophy, etc., mitochondrial permeability transition pore (mPTP) is formed and opened, which can lead to dysfunction of mitochondria and subsequently to cell death. This review summarizes the results of studies carried out by our group of the effect of astaxanthin (AST) on the functional state of rat heart mitochondria upon direct addition of AST to isolated mitochondria and upon chronic administration of AST under conditions of mPTP opening. It was shown that AST exerted a protective effect under all conditions. In addition, AST treatment was found to prevent isoproterenol-induced oxidative damage to mitochondria and increase mitochondrial efficiency. AST, a ketocarotenoid, may be a potential mitochondrial target in therapy for pathological conditions associated with oxidative damage and mitochondrial dysfunction, and may be a potential mitochondrial target in therapy for pathological conditions.

## 1. Introduction

Mitochondria are the main organelle in cells and play a key role in the normal functioning of the heart, as well as in the pathogenesis and development of various heart diseases [1]. Physiologically, mitochondrial ATP stores are consistent with changes in heart ATP consumption, and mitochondrial Ca^2+^ transport pathways that provide an increase in mitochondrial Ca^2+^ concentration mediate these changes [1]. Mitochondria are organelles that are the main source of reactive oxygen species (ROS) in the heart, as the respiratory chain activates the superoxide anion O^2−^ as part of normal respiration, and this can trigger the production of other ROS [2].

The most important precondition for the normal functioning of cells is the maintenance of the structural and functional integrity of mitochondria, since mitochondria play an important role in energy metabolism, as well as in maintaining the cellular redox state and regulation of apoptosis. Since mitochondria are the main source of ROS [2], mitochondrial dysfunction leads to oxidative stress, which can result in various disorders in the cellular activity and ultimately to their death [3]. The development of oxidative stress can be the main cause of various human diseases, such as metabolic syndrome, neurodegenerative, cardiovascular and inflammatory diseases, as well as age-related disruptions. Mitochondrial dysfunction can trigger the development of diseases associated with oxidative stress [4,5].

It is known that cardiac function is regulated by various antioxidant defense mechanisms; however, in heart disease, antioxidant protection is impaired and an increase in ROS production suppresses the ability of cells to antioxidant protection [1,4,5,6]. Recently, mitochondria-targeted antioxidants have been created to effectively combat diseases caused by ROS, for example, the MitoQ10 antioxidant of ubiquinol with a lipophilic tail of triphenylphosphonium, which accumulates 100-fold in mitochondria due to its extremely negative membrane potential [7,8]. In studies, mainly in rat models of heart disease, MitoQ10 has been shown to be useful in protecting against ischemia/reperfusion injury [9], hypertension and hypertrophy [10] and sepsis-induced cardiac dysfunction [11]. The heart has different mechanisms of antioxidant defense, however, ROS, apparently, is not suppressed in heart failure, rather it is the increase in ROS production that suppresses the antioxidant capacity [6]. Mitochondria contain several enzymes that detoxify ROS: Mn^2+^ superoxide dismutase (Mn-SOD) converts O_2_ to H_2_O_2_, and glutathione peroxidase and peroxiredoxins convert H_2_O_2_ to H_2_O [2]. In addition to adding exogenous antioxidants, strategies that enhance endogenous defense pathways are candidates for the prevention or treatment of heart failure. One of these enzymes is glutathione peroxidase, which is present in both the cytosol and mitochondria, which absorbs H_2_O_2_ and prevents the formation of hydroxyl radicals: overexpression of this enzyme in mice prevents the development of heart failure after myocardial infarction [12]. Using a similar model in rats, vitamin E supplementation was observed to also protect against heart failure, which may be related to increased catalase and glutathione peroxidase activity [13]. Disabling Mn-SOD in mitochondria also leads to dilated cardiomyopathy in mice that die within 10 days of birth [14].

There are dietary antioxidants, such as vitamins E and C, that can reduce oxidative stress [15,16], increase the protection of the mitochondrial antioxidant system [17] and, as a result, prevent the development of cardiovascular disease. Among the dietary antioxidants are carotenoids, which are divided into carotenes and xanthophyll. The group of carotenes includes β-carotene and lycopene, and the group of xanthophylls contains lutein, canthaxanthin, zeaxanthin, violaxanthin, capsorubin and astaxanthin [18,19]. Astaxanthin (AST) is of the greatest interest for research because it is obtained from natural sources as an ester of fatty acids or as a conjugate of proteins in food [3].

In this review, we present our findings, which shed light on the function of AST in heart failure and we hypothesized that mitochondria may be the target of the protective effect of AST.

## 2. The Biological Role of Astaxanthin

Astaxanthin (AST) was first isolated from lobsters [20]. AST belongs to a broad class of chemical compounds known as terpenes and is classified as xanthophyll because it has two additional oxygen atoms on each of the six-membered rings compared to beta-carotene [21,22]. AST is present in most red-colored aquatic organisms and has been found in the tissues of a variety of fish, shrimp, birds and plants. The red color of salmon meat is due to the presence of AST in it. Content varies both between species and between individuals, as it is highly dependent on diet and living conditions. AST and other chemically related asta-carotenoids have also been found in a number of lichen species in the arctic zone [23]. The *Haematococcus pluvialis* alga is an essential source of AST for industrial production. Under normal conditions, it has a green color, but with a decrease in the amount of food, it goes into a dormant state and begins to produce AST to protect against ultraviolet radiation and oxidation [24]. In nature, AST can be present not only in free form, but also in the form of mono- and di-esters. In Antarctic krill, up to 65% of AST is contained in the form of diester, in algae up to 70% in the form of monoester and in red yeast—100% in free form [25].

**Figure 1:**
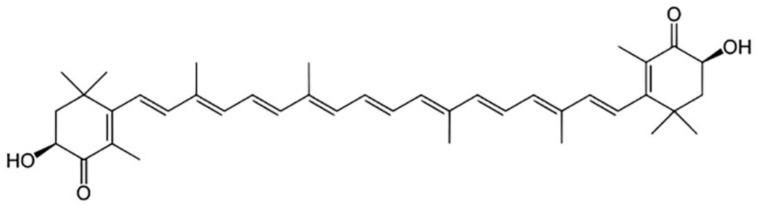
The structure formula of astaxanthin.

AST affects the biochemical processes occurring in almost all organs and tissues of a human. Among the well-known properties of AST, its antioxidant and anti-inflammatory properties can be noted. Whereas AST has such a molecular structure (Figure 1) containing hydroxyl and keto moieties on each ionone ring, it therefore exhibits high antioxidant properties [26,27].

The activity of AST as an antioxidant is 10 times higher than that of zeaxanthin, lutein, canthaxanthin and beta-carotene, and 100 times higher than that of alpha-tocopherol. Due to its molecular structure, AST remains both inside and outside the cell membrane, therefore it provides cells with protection against oxidative damage caused by various mechanisms; traps free radicals to prevent chain reactions; preserves the membrane structure by inhibiting lipid peroxidation; enhances the functions of the immune system and participates in the regulation of gene expression.

It is known that, due to increased ROS production and mitochondrial leakage, mitochondrial dysfunction can induce the expression of pro-inflammatory cytokines, increase the sensitivity of cells to inflammatory signaling, induce a molecular pattern associated with damage and activate the inflammasomes [28]. It has been shown that AST can have a prophylactic effect in degenerative pathological conditions caused by oxidative stress. For example, in a mouse model of Alzheimer’s disease, AST in the form of an ester with docosahexaenoic acid reduced oxidative stress and the inflammasome activation [29]. AST can increase the stability of cell membranes by preventing the penetration of substances that promote lipid peroxidation through the lipid layer [30] and can provide additional protection against damage caused by free radicals [31]. The antioxidant effect of AST is a clinically significant, especially in people who are susceptible to oxidative stress, such as smokers and overweight people [32]. Moreover, AST is able to inhibit the induction of inflammation in biological systems. AST has been shown to reduce bacterial load and gastric inflammation in *H. pylori*-infected mice [33]. In addition, AST reduced inflammation, a biomarker of oxidative DNA damage, thereby enhancing the immune response in young healthy adult women [34].

AST is also known to be able to reduce the oxidative stress caused by hyperglycemia in the β-cells of the pancreas, and AST has been observed to improve glucose and serum insulin levels. Therefore, AST is able to protect β-cells of the pancreas from glucose toxicity [35]. It was also shown that during the restoration of lymphocyte dysfunction associated with diabetic rats, AST proved to be a good immunological agent [36]. AST increased the total number of T and B cells relative to placebo effect, as well as the cytotoxic activity of natural killer cells, which indicates its effect on the immune system of the organism [34]. AST has shown antitumor activity in various types of cancers. Thus, it suppressed the growth of fibrosarcoma, breast and prostate cancer cells, and embryonic fibroblasts [37]. Astaxanthin inhibited cell death and proliferation in breast tumors in male and female rats and mice induced by chemical means [38,39]. There is evidence of a positive effect of AST on deceleration of the degradation of cognitive functions caused by age-related changes in people with dementia [40].

**Figure 2:**
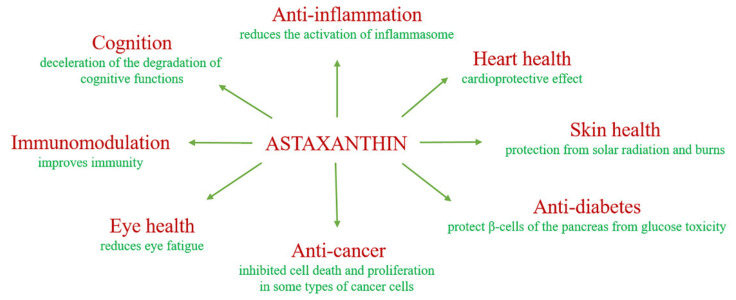
Scheme of the biological benefits of AST.

Moreover, there was also a positive trend in the course of the disease, an improvement in the ability to remember in mice [41]. In addition, AST has a positive effect on smoothing wrinkles, moisturizing and skin tone, its elasticity, smoothness, puffiness and age spots [42]. AST also has a positive effect on visual acuity even in healthy people, reduces eye fatigue and in senile farsightedness, AST has a positive effect on vision due to the improvement of the contractility of the papillary [43]. Figure 2 shows some of the benefits of astaxanthin.

## 3. Astaxanthin and Mitochondrial Permeability Transition Pore Opening (mPTP)

### 3.1. What Is mPTP?

Mitochondrial permeability transition pore (mPTP) is a mitochondrial Ca^2+^-dependent cyclosporine A (CsA)-sensitive pore that is formed by a complex of proteins and is a channel that passes through the outer and inner membranes of the mitochondria. This channel is considered a pore that changes the permeability of the mitochondrial membrane [44]. Until now, the final composition of mPTP has not been established. Among the regulator components of the pores, the voltage-dependent anion channel (VDAC) and the translocator protein (TSPO), located in the outer mitochondrial membrane, are distinguished. Adenine nucleotide translocase (ANT) in the inner membrane, cyclophilin D (CyP-D) and a phosphate transporter in the matrix [45]. It has been shown that VDAC and ANT are not structural components of mPTP [46,47] however, these proteins are considered regulators of mPTP. Recently, in our laboratory, a protein in the nonsynaptic mitochondria of the rat brain was identified as 2’,3’-cyclonucleotide-3’-phosphodiesterase (CNPase) [48]. We have shown that CNPase is involved in the regulation of mPTP opening [49]. In addition, we found that an ADAP1, a brain-specific protein (known recently as p42^IP4^ or Centaurin-α1 is also implicated in the function of mPTP [50]. Moreover, CNPase co-localizes with CyP-D, VDAC, ANT, ADAP1 and α-tubulin [51]. Subunit c, mitochondrial (N, N-dicyclohexylcarbodiimide DCCD-binding proteolipid) [52], also known as subunit 9 F0c, forms in cooperation with subunit α, proton channel of FoF_1_-ATPase [53]. We have recently shown that the subunit c FoF_1_-ATPase can be a structural and/or regulatory component of the mPTP complex, the activity of which can be modulated by Ca^2+^-dependent phosphorylation [54].

mPTP is a nonselective channel that plays a significant role in Ca^2+^ exchange between mitochondria and the environment [55]. Ca^2+^ influx and efflux from mitochondria occur in different ways. So, into the matrix, Ca^2+^ passes through the Ca^2+^-uniporter—the voltage-dependent Ca^2+^ channel of the inner mitochondrial membrane and leaves the matrix through Na^+^/Ca^2+^—and H^+^/Ca^2+^ exchangers or through mPTP [55,56]. Ca^2+^ initiating the opening of mPTP plays a regulatory role in the functioning of mPTP, i.e., activates its opening from the side of the matrix, but also blocks it from the outside of the mitochondrial membrane.

Martin Crompton was the first to acknowledge that the opening of mPTP may cause heart damage during reperfusion after a period of ischemia [57,58]. Subsequent studies using isolated cardiac myocytes [59,60] and perfused Langendorff hearts [61,62] directly showed that the mPTP opening does occur with such reperfusion injury, and that preventing the opening of the mPTP provides protection against reperfusion injury. The results of many investigations have shown the central role of mPTP in reperfusion injury and its importance as a pharmacological target for cardioprotection [63,64,65,66]. Due to its central role in reperfusion injury, mPTP has become an obvious target for cardioprotection.

An increase in mitochondrial matrix of Ca^2+^ alone may not be sufficient to trigger the opening of mPTP, and additional factors such as oxidative stress, adenine nucleotide depletion, increased phosphate concentrations and mitochondrial membrane depolarization are considered critical. Indeed, such factors, and especially oxidative stress, may be more important than the increase in Ca^2+^ for mPTP opening seen under conditions such as ischemia/reperfusion [64,67,68,69].

### 3.2. The Involvement of AST in the Protection of Mitochondria from Ca^2+^-Induced Oxidative Stress

AST is known to reduce oxidative stress and maintain mitochondrial integrity. Wolf and coauthors showed that AST improves mitochondrial function by protecting mitochondrial redox balance [70]. Interestingly, AST significantly reduced physiologically occurring oxidative stress and maintained mitochondria in a more reduced state even after H_2_O_2_ stimulation. It also prevented a drop in membrane potential (Δψm) and increased mitochondrial oxygen consumption. AST can prevent mitochondrial dysfunction by penetrating and localizing in mitochondria [71,72]. In our studies, we studied the effect of AST on the opening of mPTP both when directly added and when it is chronically administered to rats for two weeks orally. Park and coauthors showed that AST treatment increased mitochondrial content, ATP production and the activity of respiratory chain complexes [73]. It is known that the respiratory control index (RCI) indicates the effectiveness of mitochondria in stimulating oxidative phosphorylation and the relationship between O_2_ consumption and ATP production. The addition of AST (5 μM) to rat heart mitochondria increased RCI and the ratio of P/O [74]. We have demonstrated that Ca^2+^-induced mPTP opening is delayed at 5 μM AST in isolated RHM. AST was able to suppress Ca^2+^-induced Ca^2+^ release and Δψm dissipation and increase CRC. To test the inhibitory effect of AST, we examined another parameter that characterizes the opening of mPTP, Ca^2+^-induced mitochondrial swelling. The addition of Ca^2+^ at a threshold concentration to the mitochondrial suspension caused a decrease in light scattering, which indicates swelling of mitochondria. Thus, the addition of AST to mitochondria increased the Ca^2+^ capacity in the RHM, while the rate of mitochondrial swelling decreased. AST prevented mitochondrial swelling and delayed Ca^2+^ release from RHM when AST was added to mitochondria [74]. This result demonstrates the involvement of AST in mPTP functioning and is consistent with literature data showing an inhibitory effect of AST on oxidative stress-induced mitochondrial dysfunction [75].

We obtained similar results on the effect of AST on the functional state of mitochondria after chronic oral administration of AST to rats. The results of the study suggested that AST is able to improve the functional state of RHM, increasing the ratio of RCI and P/O both with the addition of AST to RHM and with AST administration. AST is an antioxidant that is permeable to mitochondria [72] and can effectively prevent oxidative stress. AST increases the resistance of RHM to Ca^2+^-dependent stress; it can be assumed that, after further research, this antioxidant can be considered an effective tool for improving the functioning of the heart muscle in general, both under normal conditions and under clinical conditions.

### 3.3. The Effect of Chronic Administration of AST on the Change in the Content of Proteins-Regulators of mPTP

In our studies, we have shown that AST can alter the expression of mPTP-regulator proteins. The role of translocator protein (TSPO) previously named peripheral benzodiazepine receptor, in the heart is not fully understood; however, this protein is known to be involved in the pathophysiology of heart disease, and its ligands improve cardiac function, which makes TSPO a potential target for the therapy of cardiovascular diseases [76]. In the heart, the level of TSPO varies depending on stressful conditions; in chronic stress, its level decreases, and in acute stress, it increases [76]. We showed that the TSPO level in isolated RHM decreased, probably due to the inhibitory effect of AST administration. TSPO forms a multimeric complex with VDAC, another mPTP regulator [77]. It is known that VDAC regulates the rate of Ca^2+^ penetration into the intermembrane space [46], thereby participating in the regulation of mPTP. A decrease in VDAC content in RHM isolated from rats treated with AST suggests a decrease in the rate of Ca^2+^ influx and, therefore, a slower opening of mPTP.

CyP-D is a mitochondrial matrix protein that is considered a structural component and regulator of mPTP, as well as an important mediator of mPTP. mPTP regulated by CyP-D is required for proper regulation of mitochondrial metabolism [78]. Loss of CyP-D does not prevent mPTP from opening but increases the Ca^2+^ load required to open [79]. CyP-D directly binds the lateral leg of ATP synthase and alters its activity [80] and also controls the assembly of the electron transport chain, making it a central node for the control of mitochondrial function [81]. Moreover, the CyP-D interaction reduces the rate of ATP synthesis and hydrolysis to modulate energy production and necrotic cell death [82]. Decreased CyP-D content in RHM isolated from AST-treated rats may result in increased Ca^2+^ loading and slower mPTP opening [74]. Subunit *c* of F_o_ sector of F_o_F_1_-ATPase plays a critical role in the formation of the Ca^2+^-induced mPTP channel [54,83,84]. In the presence of a threshold value of [Ca^2+^], the dephosphorylated subunit *c* has the ability to stimulate the opening of mPTP and induce mitochondrial swelling, as well as to reduce the ability to uptake Ca^2+^ and Δψm. In mitochondria, when mPTP is opened, the level of subunit *c* decreases [54]. In RHM isolated from rats treated with AST, the content of subunit *c* increased, which could contribute to an increase in Ca^2+^ capacity and a slowdown in mitochondrial swelling [85].

## 4. Astaxanthin Administration and Heart Failure

Our further research was aimed at studying the effect of AST administration on the structure of the heart tissue, the functional state of the RHM, the activity of the respiratory complexes and the levels of the main subunits of the ETC complexes in heart failure caused by isoproterenol (ISO). For this purpose, four groups of rats were studied. The rats of the first group were the control; the rats from the second group were orally treated with AST. The rats of the third group were injected with ISO to cause acute heart failure by the method adopted in the world scientific community [86]. The rats of the fourth group were orally treated with AST and two weeks later, they were injected twice with ISO.

The results obtained by histological analysis suggest that the use of AST significantly reduced both degeneration and post ischemic edema of the muscle fibers of the heart, and the degree of fibrotic myocardial damage after acute heart failure caused by ISO. The data obtained using digital bioimaging of transmural histotopograms of left ventricle of the studied groups allowed us to conclude a significant decrease in the degree of fibrosis of subendocardial lesions of the heart, which indicates a protective effect from the administration of AST [85].

The electron transport chain (ETC), which consists of transmembrane protein complexes (I–IV), is located in the inner membrane of mitochondria. For proper operation, the complexes are assembled into a specially tuned supercomplex, which, together with CV, become the basis for the production of ATP during oxidative phosphorylation. It should be noted that defects in the respiratory complexes and ATP synthase affect the function of mitochondria. [87,88]. We showed that the level of the main subunits of the respiratory chain complexes in RHM in rats injected with ISO decreased, which indicates the development of mitochondrial damage in rats. AST abolished the effect of ISO and increased the content of subunits in the RHM. Complexes III, IV and I are involved in the pumping of electrons and the subsequent generation of a directed proton gradient across the inner mitochondrial membrane. In addition, the degree of damage to mitochondrial proteins increases in various pathologies, which leads to a decrease in the efficiency of mitochondria and the production of cellular energy [89]. Heart failure can suppress the expression of ETC subunits and reduce their activity [1]. We found that ISO reduced the activity of complexes I, II, IV and ATP synthase in RHM, while AST eliminated the effect of ISO and increased their activity. It is generally accepted that the main subunits of the respiratory chain complexes have a significant effect on the efficiency of mitochondria. It should be noted that a decrease in the level of subunits of these complexes can cause a decrease in the activity of the complexes and mitochondrial respiration in RHM in rats injected with ISO.

Cardiolipin (CL) is a phospholipid that is involved in the development of structural integrity and enzymatic activity in the complexes of the respiratory chain of mitochondria. CL plays an important role in mitochondrial bioenergy by stimulating the activity of key proteins of inner mitochondrial membrane, namely several anionic transporters and some complexes of ETC [90], and is a major phospholipid involved in maintaining mitochondrial function and myocardial health [91]. Loss of CL in heart disease increases the production of ROS and enhances the peroxidation of cardiolipin, which leads to dysfunction of mitochondria and, ultimately, to the death of cardiomyocytes [92]. There are specific binding sites for CL with Complex I [93], Complex III [94], Complex IV [95] and Complex II [96]. We noticed that a decrease in the level of CL in RHM isolated from rats that injected with ISO can reduce the expression of the main subunits of the ETC complexes and, therefore, impair the functional state of mitochondria. In addition, CL is involved in the functioning of mPTP [97], possibly for this reason, the Ca^2+^ capacity in RHM in rats injected with ISO decreased, which accelerated the opening of mPTP and could increase the rate of mitochondrial swelling. AST improved the functional state of RHM in ISO-treated rats, while CL levels increased, resulting in increased RCI and Ca^2+^ capacity and slowed the rate of mitochondrial swelling.

Antioxidants trigger a defense mechanism that breaks down harmful ROS and inhibits lipid peroxidation. In this case, enzymes neutralizing free radicals such as catalase, glutathione peroxidase and superoxide dismutase help the restore of protective antioxidant system and inhibit ROS production [98]. In our study, we noticed a decrease in the level of Mn-SOD-2 in RHM in rats that were injected with ISO. The administration of AST significantly increased the level of Mn-SOD-2 in RHM rats injected with ISO. AST provided protection for cardiac tissue from oxidative damage.

ATP synthase plays a central role in maintaining the energy state of cells and the respiratory function of mitochondria [99]. A decrease in activity of ATP synthase strongly affects mitochondrial respiration and, consequently, cardiac activity, since disturbances in mitochondrial energy are involved in the development of various heart pathologies [100]. Complex V (CV) consists of two functional parts: Fo and F_1_. The Fo complex contains transmembrane subunits that transport protons from the intermembrane space, and F_1_ is a peripheral complex in the matrix that binds to nucleotides and inorganic phosphate to synthesize ATP [101,102]. ATP synthase is known to catalyze the final step of oxidative phosphorylation to provide energy in the form of ATP. Changes at this stage can decisively affect mitochondrial respiration and, therefore, the work of the heart. It is known that the contractile ability of the heart is strongly dependent on mitochondria and that a decrease in the level of myocardial ATP is a key sign of heart failure. In mitochondria, subunit alpha (ATP5A) is the part of F_1_ sector; subunit *c* (ATP5G) and *b* (ATP5F1) are the parts of Fo sector of ATP synthase [99]. We showed that ISO reduced CV activity in RHM, while AST abolished the ISO effect and increased CV activity. Under these conditions, the level of subunits *c* and *b* decreased in RHM after BNE in rats injected with ISO, while AST eliminated the effect of ISO and increased the level of all CV subunits. Chronic administration of AST increased the level of subunits of the respiratory chain complexes, ATP synthase, which suggests that AST prevents oxidative damage by increasing mitochondrial efficiency.

## 5. Summary

AST is able to improve the functional state of RHM by increasing RCI and P/O ratio both with the administration of AST to rats and with direct addition of AST to isolated mitochondria. AST, a dietary carotenoid, can penetrate to the mitochondria and inhibit the mPTP opening. The AST administration and direct addition of AST to mitochondria can delay Ca^2+^-induced Ca^2+^ release.

AST administration enhanced the activity of the respiratory chain and ATP synthase complexes in RHM exposed to ISO injection. The AST administration increased the level of subunits of the respiratory chain complexes and ATP synthase in intact RHM samples, suggesting that AST prevents oxidative damage and increases mitochondrial efficiency. CyP-D regulates mitochondrial oxidative phosphorylation. The AST administration decreased the content of CyP-D and increased the levels of ANT, subunits of the respiratory chain complexes, and ATP synthase subunits in the RHM after the injection of ISO, which indicates an improvement in the functional state of RHM and mitochondrial respiration. It may be the reason for the increased activity of the complexes of the respiratory chain and ATP synthase. The AST treatment led to an increase in the level of Mn-SOD-2 in the RHM in rats that were injected with ISO, thus protecting against oxidative damage. The administration of AST inhibited the elimination of CL, which plays an important role in the regulation of membrane integrity and the activity of the respiratory chain complexes.

**Figure 3:**
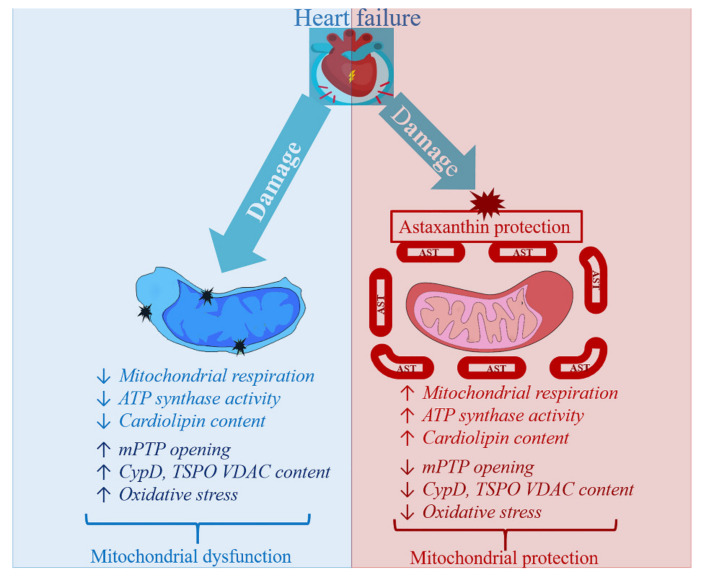
Scheme of the AST effects in Heart failure. Up arrow indicates increase or enhancement, down arrow means decrease or reduction.

AST has a protective effect in RHM and can be considered an effective drug for improving cardiac muscle function, both under normal and clinical conditions. The mechanisms by which AST acts in mitochondria need to be determined. However, based on the above, there is no doubt that AST exerts its effect through the mitochondria (Figure 3). We concluded that AST may be a potential target in mitochondria in therapy for pathological conditions associated with oxidative damage and mitochondrial dysfunction.

## Data Availability

No new data were created or analyzed in this study. Data sharing is not applicable to this article.

## References

[1] Griffiths E.J. (2011). Mitochondria and Heart Disease. Adv. Exp. Med. Biol..

[2] Murphy M.P. (2009). How mitochondria produce reactive oxygen species. Biochem. J..

[3] Kim S.H., Kim H. (2018). Inhibitory Effect of Astaxanthin on Oxidative Stress-Induced Mitochondrial Dysfunction—A Mini-Review. Nutrients.

[4] Bullon P., Newman H.N., Battino M. (2014). Obesity, diabetes mellitus, atherosclerosis and chronic periodontitis: A shared pathology via oxidative stress and mitochondrial dysfunction?. Periodontol. 2000.

[5] Hernandez-Aguilera A., Rull A., Rodriguez-Gallego E., Riera-Borrull M., Luciano-Mateo F., Camps J., Menendez J.A., Joven J. (2013). Mitochondrial dysfunction: A basic mechanism in inflammation-related non-communicable diseases and therapeutic opportunities. Mediators Inflamm..

[6] Tsutsui H., Kinugawa S., Matsushima S. (2008). Oxidative Stress and Mitochondrial DNA Damage in Heart Failure. Circ. J..

[7] James A.M., Cocheme H.M., Smith R.A., Murphy M.P. (2005). Interactions of mitochondria-targeted and untargeted ubiquinones with the mitochondrial respiratory chain and reactive oxygen species. Implications for the use of exogenous ubiquinones as therapies and experimental tools. J. Biol. Chem..

[8] Murphy M.P., Smith R.A. (2007). Targeting Antioxidants to Mitochondria by Conjugation to Lipophilic Cations. Annu. Rev. Pharmacol. Toxicol..

[9] Adlam V.J., Harrison J.C., Porteous C.M., James A.M., Smith R.A., Murphy M.P., Sammut I. (2005). Targeting an antioxidant to mitochondria decreases cardiac ischemia-reperfusion injury. FASEB J..

[10] Graham D., Huynh N.N., Hamilton C.A., Beattie E., Smith R.A., Cochemeé H.M., Murphy M.P., Dominiczak A.F. (2009). Mitochondria-Targeted Antioxidant MitoQ 10 Improves Endothelial Function and Attenuates Cardiac Hypertrophy. Hypertension.

[11] Supinski G.S., Murphy M.P., Callahan L.A. (2009). MitoQ administration prevents endotoxin-induced cardiac dysfunction. Am. J. Physiol. Regul. Integr. Comp. Physiol..

[12] Shiomi T., Tsutsui H., Matsusaka H., Murakami K., Hayashidani S., Ikeuchi M., Wen J., Kubota T., Utsumi H., Takeshita A. (2004). Overexpression of Glutathione Peroxidase Prevents Left Ventricular Remodeling and Failure After Myocardial Infarction in Mice. Circulation.

[13] Hill M.F., Palace V.P., Kaur K., Kumar D., Khaper N., Singal P.K. (2005). Reduction in oxidative stress and modulation of heart failure subsequent to myocardial infarction in rats. Exp. Clin. Cardiol..

[14] Li Y., Huang T.-T., Carlson E.J., Melov S., Ursell P.C., Olson J.L., Noble L.J., Yoshimura M.P., Berger C.N., Chan P.H. (1995). Dilated cardiomyopathy and neonatal lethality in mutant mice lacking manganese superoxide dismutase. Nat. Genet..

[15] Carpenter K.L., Kirkpatrick P.J., Weissberg P.L., Challis I.R., Dennis I.F., Freeman M.A., Mitchinson M.J. (2003). Oral alpha-tocopherol supplementation inhibits lipid oxidation in established human atherosclerotic lesions. Free Radic. Res..

[16] Ellingsen I., Seljeflot I., Arnesen H., Tonstad S. (2009). Vitamin C consumption is associated with less progression in carotid intima media thickness in elderly men: A 3-year intervention study. Nutr. Metab. Cardiovasc. Dis..

[17] Poljsak B., Suput D., Milisav I. (2013). Achieving the balance between ros and antioxidants: When to use the synthetic antioxi-dants. Oxid. Med. Cell Longev..

[18] Jackson H., Braun C.L., Ernst H. (2008). The Chemistry of Novel Xanthophyll Carotenoids. Am. J. Cardiol..

[19] McNulty H.P., Byun J., Lockwood S.F., Jacob R.F., Mason R.P. (2007). Differential effects of carotenoids on lipid peroxidation due to membrane interactions: X-ray diffraction analysis. Biochim. Biophys. Acta.

[20] Chang J.-H., Chen Y., Holland D., Grabowski J. (2010). Estimating spatial distribution of American lobster Homarus americanus using habitat variables. Mar. Ecol. Prog. Ser..

[21] Choi S., Koo S. (2005). Efficient Syntheses of the Keto-carotenoids Canthaxanthin, Astaxanthin, and Astacene. J. Org. Chem..

[22] Margalith P.Z. (1999). Production of ketocarotenoids by microalgae. Appl. Microbiol. Biotechnol..

[23] Ambati R.R., Phang S.-M., Ravi S., Aswathanarayana R.G. (2014). Astaxanthin: Sources, Extraction, Stability, Biological Activities and Its Commercial Applications—A Review. Mar. Drugs.

[24] Spiller G.A., Dewell A. (2003). Safety of an Astaxanthin-Rich Haematococcus pluvialisAlgal Extract: A Randomized Clinical Trial. J. Med. Food.

[25] Vitale G.A., Coppola D., Palma Esposito F., Buonocore C., Ausuri J., Tortorella E., De Pascale D. (2020). Antioxidant Molecules from Marine Fungi: Methodologies and Perspectives. Antioxidants (Basel).

[26] Hussein G., Sankawa U., Goto H., Matsumoto K., Watanabe H. (2006). Astaxanthin, a Carotenoid with Potential in Human Health and Nutrition. J. Nat. Prod..

[27] Liu X.B., Osawa T. (2007). Cis astaxanthin and especially 9-cis astaxanthin exhibits a higher antioxidant activity in vitro compared to the all-trans isomer. Biochem. Biophys. Res. Commun..

[28] Novak E.A., Mollen K.P. (2015). Mitochondrial dysfunction in inflammatory bowel disease. Front. Cell Dev. Biol..

[29] Che H., Li Q., Zhang T., Wang D., Yang L., Xu J., Yanagita T., Xue C., Chang Y., Wang Y. (2018). Effects of Astaxanthin and Docosahexaenoic-Acid-Acylated Astaxanthin on Alzheimer’s Disease in APP/PS1 Double-Transgenic Mice. J. Agric. Food Chem..

[30] Wisniewska A., Subczynski W.K. (1998). Effects of polar carotenoids on the shape of the hydrophobic barrier of phospholipid bilayers. Biochim. Biophys. Acta.

[31] Edge R., Gaikwad P., Navaratnam S., Rao B.S., George Truscott T. (2010). Reduction of oxidized guanosine by dietary carotenoids: A pulse radiolysis study. Arch. Biochem. Biophys..

[32] Yuan J.-P., Peng J., Yin K., Wang J.-H. (2011). Potential health-promoting effects of astaxanthin: A high-value carotenoid mostly from microalgae. Mol. Nutr. Food Res..

[33] Rao A.R., Reddy A.H., Aradhya S.M. (2010). Antibacterial properties of spirulina platensis, haematococcus pluvialis, botryococcus braunii micro algal extracts. Curr. Trends Biotechnol. Pharm..

[34] Park J.S., Chyun J.H., Kim Y.K., Line L.L., Chew B.P. (2010). Astaxanthin decreased oxidative stress and inflammation and enhanced immune response in humans. Nutr. Metab. (Lond.).

[35] Uchiyama K., Naito Y., Hasegawa G., Nakamura N., Takahashi J., Yoshikawa T. (2002). Astaxanthin protects beta-cells against glucose toxicity in diabetic db/db mice. Redox Rep..

[36] Otton R., Marin D.P., Bolin A.P., de Cássia Macedo dos Santos R., Polotow T.G., Sampaio S.C., Paes de Barros M. (2010). Astaxanthin ameliorates the redox imbalance in lymphocytes of experimental diabetic rats. Chem. Biol. Interact..

[37] Palozza P., Torelli C., Boninsegna A., Simone R., Catalano A., Mele M.C., Picci N. (2009). Growth-inhibitory effects of the astaxanthin-rich alga Haematococcus pluvialis in human colon cancer cells. Cancer Lett..

[38] Nakao R., Nelson O.L., Park J.S., Mathison B.D., Thompson P.A., Chew B.P. (2010). Effect of dietary astaxanthin at different stages of mammary tumor initiation in BALB/c mice. Anticancer Res..

[39] Prabhu P.N., AshokKumar P., Sudhandiran G. (2009). Antioxidative and antiproliferative effects of astaxanthin during the initiation stages of 1,2-dimethyl hydrazine-induced experimental colon carcinogenesis. Fundam. Clin. Pharmacol..

[40] Satoh A., Tsuji S., Okada Y., Murakami N., Urami M., Nakagawa K., Ishikura M., Katagiri M., Koga Y., Shirasawa T. (2009). Preliminary Clinical Evaluation of Toxicity and Efficacy of A New Astaxanthin-rich Haematococcus pluvialis Extract. J. Clin. Biochem. Nutr..

[41] Zhang X., Pan L., Wei X., Gao H., Liu J. (2007). Impact of astaxanthin-enriched algal powder of Haematococcus pluvialis on memory improvement in BALB/c mice. Environ. Geochem. Health.

[42] Tominaga K., Hongo N., Karato M., Yamashita E. (2012). Cosmetic benefits of astaxanthin on humans subjects. Acta Biochim. Pol..

[43] D’Orazio N., Gemello E., Gammone M.A., De Girolamo M., Ficoneri C., Riccioni G. (2012). Fucoxantin: A Treasure from the Sea. Mar. Drugs.

[44] Halestrap A.P. (2009). What is the mitochondrial permeability transition pore?. J. Mol. Cell. Cardiol..

[45] Tsujimoto Y., Shimizu S. (2007). Role of the mitochondrial membrane permeability transition in cell death. Apoptosis.

[46] Hurst S., Hoek J., Sheu S.-S. (2017). Mitochondrial Ca(2+) and regulation of the permeability transition pore. J. Bioenerg. Biomembr..

[47] Morciano G., Giorgi C., Bonora M., Punzetti S., Pavasini R., Wieckowski M.R., Campo G., Pinton P. (2015). Molecular identity of the mitochondrial permeability transition pore and its role in ischemia-reperfusion injury. J. Mol. Cell. Cardiol..

[48] Azarashvili T., Krestinina O., Galvita A., Grachev D., Baburina Y., Stricker R., Reiser G. (2014). Identification of phosphorylated form of 2’, 3’-cyclic nucleotide 3’-phosphodiesterase (cnpase) as 46 kda phosphoprotein in brain non-synaptic mitochondria overloaded by calcium. J. Bioenerg. Biomembr..

[49] Azarashvili T., Krestinina O., Galvita A., Grachev D., Baburina Y., Stricker R., Evtodienko Y., Reiser G. (2009). Ca2+-dependent permeability transition regulation in rat brain mitochondria by 2’,3’-cyclic nucleotides and 2’,3’-cyclic nucleotide 3’-phosphodiesterase. Am. J. Physiol Cell Physiol..

[50] Galvita A., Grachev D., Azarashvili T., Baburina Y., Krestinina O., Stricker R., Reiser G. (2009). The brain-specific protein, p42(iP4)(ADAP 1) is localized in mitochondria and involved in regulation of mitochondrial Ca2+. J. Neurochem..

[51] Baburina Y., Azarashvili T., Grachev D., Krestinina O., Galvita A., Stricker R., Reiser G. (2015). Mitochondrial 2′, 3′-cyclic nucleotide 3′-phosphodiesterase (CNP) interacts with mPTP modulators and functional complexes (I–V) coupled with release of apoptotic factors. Neurochem. Int..

[52] Célis H. (1980). 1-Butanol extracted proteolipid. Proton conducting properties. Biochem. Biophys. Res. Commun..

[53] Wittig I., Schägger H. (2008). Structural organization of mitochondrial ATP synthase. Biochim. Biophys. Acta.

[54] Azarashvili T., Odinokova I., Bakunts A., Ternovsky V., Krestinina O., Tyynelä J., Saris N.-E.L. (2014). Potential role of subunit c of F0F1-ATPase and subunit c of storage body in the mitochondrial permeability transition. Effect of the phosphorylation status of subunit c on pore opening. Cell Calcium.

[55] Gunter T.E., Yule D.I., Gunter K.K., Eliseev R.A., Salter J.D. (2004). Calcium and mitochondria. FEBS Lett..

[56] Crompton M., Barksby E., Johnson N., Capano M. (2002). Mitochondrial intermembrane junctional complexes and their involvement in cell death. Biochimie.

[57] Crompton M., Costi A. (1988). Kinetic evidence for a heart mitochondrial pore activated by Ca2+, inorganic phosphate and oxidative stress. A potential mechanism for mitochondrial dysfunction during cellular Ca2+ overload. Eur. J. Biochem..

[58] Crompton M., Costi A., Hayat L. (1987). Evidence for the presence of a reversible Ca2+-dependent pore activated by oxidative stress in heart mitochondria. Biochem. J..

[59] Nazareth W., Yafei N., Crompton M. (1991). Inhibition of anoxia-induced injury in heart myocytes by cyclosporin A. J. Mol. Cell. Cardiol..

[60] Leyssens A., Nowicky A.V., Patterson L., Crompton M., Duchen M.R. (1996). The relationship between mitochondrial state, ATP hydrolysis, [Mg2+]i and [Ca2+]i studied in isolated rat cardiomyocytes. J. Physiol..

[61] Griffiths E.J., Halestrap A.P. (1995). Mitochondrial non-specific pores remain closed during cardiac ischaemia, but open upon reperfusion. Biochem. J..

[62] Griffiths E.J., Halestrap A. (1993). Protection by Cyclosporin A of Ischemia/Reperfusion-Induced Damage in Isolated Rat Hearts. J. Mol. Cell. Cardiol..

[63] Di Lisa F., Bernardi P. (2006). Mitochondria and ischemia–reperfusion injury of the heart: Fixing a hole. Cardiovasc. Res..

[64] Halestrap A.P., Clarke S.J., Javadov S.A. (2004). Mitochondrial permeability transition pore opening during myocardial reperfusion—A target for cardioprotection. Cardiovasc. Res..

[65] Halestrap A.P., Pasdois P. (2009). The role of the mitochondrial permeability transition pore in heart disease. Biochim. Biophys. Acta.

[66] Yellon D.M., Hausenloy D.J. (2007). Myocardial Reperfusion Injury. N. Engl. J. Med..

[67] Halestrap A.P., McStay G.P., Clarke S.J. (2002). The permeability transition pore complex: Another view. Biochimie.

[68] Juhaszova M., Wang S., Zorov D.B., Nuss H.B., Gleichmann M., Mattson M.P., Sollott S.J. (2008). The identity and regulation of the mitochondrial permeability transition pore: Where the known meets the unknown. Ann. N. Y. Acad. Sci..

[69] Kim J.S., Jin Y., Lemasters J.J. (2006). Reactive oxygen species, but not ca2+ overloading, trigger ph- and mitochondrial permeability transition-dependent death of adult rat myocytes after ischemia-reperfusion. Am. J. Physiol. Heart Circ. Physiol..

[70] Wolf A.M., Asoh S., Hiranuma H., Ohsawa I., Iio K., Satou A., Ishikura M., Ohta S. (2010). Astaxanthin protects mitochondrial redox state and functional integrity against oxidative stress. J. Nutr. Biochem..

[71] Kuroki T., Ikeda S., Okada T., Maoka T., Kitamura A., Sugimoto M., Kume S. (2013). Astaxanthin ameliorates heat stress-induced impairment of blastocyst development in vitro:--astaxanthin colocalization with and action on mitochondria. J. Assist. Reprod. Genet..

[72] Zhang Z.-W., Xu X.-C., Liu T., Yuan S. (2016). Mitochondrion-Permeable Antioxidants to Treat ROS-Burst-Mediated Acute Diseases. Oxid. Med. Cell. Longev..

[73] Park J.S., Mathison B.D., Hayek M.G., Zhang J., Reinhart G.A., Chew B.P. (2013). Astaxanthin modulates age-associated mi-tochondrial dysfunction in healthy dogs. J. Anim. Sci..

[74] Baburina Y., Krestinin R., Odinokova I., Sotnikova L., Kruglov A., Krestinina O. (2019). Astaxanthin Inhibits Mitochondrial Permeability Transition Pore Opening in Rat Heart Mitochondria. Antioxidants (Basel).

[75] Wu Y., Shamoto-Nagai M., Maruyama W., Osawa T., Naoi M. (2016). Phytochemicals prevent mitochondrial membrane permeabilization and protect SH-SY5Y cells against apoptosis induced by PK11195, a ligand for outer membrane translocator protein. J. Neural Transm. (Vienna).

[76] Morin D., Musman J., Pons S., Berdeaux A., Ghaleh B. (2016). Mitochondrial translocator protein (TSPO): From physiology to cardioprotection. Biochem. Pharmacol..

[77] McEnery M.W., Snowman A.M., Trifiletti R.R., Snyder S.H. (1992). Isolation of the mitochondrial benzodiazepine receptor: Association with the voltage-dependent anion channel and the adenine nucleotide carrier. Proc. Natl. Acad. Sci. USA.

[78] Elrod J.W., Molkentin J.D. (2013). Physiologic Functions of Cyclophilin D and the Mitochondrial Permeability Transition Pore. Circ. J..

[79] Basso E., Fante L., Fowlkes J., Petronilli V., Forte M.A., Bernardi P. (2005). Properties of the Permeability Transition Pore in Mitochondria Devoid of Cyclophilin D. J. Biol. Chem..

[80] Giorgio V., Bisetto E., Soriano M.E., Dabbeni-Sala F., Basso E., Petronilli V., Forte M.A., Bernardi P., Lippe G. (2009). Cy-clophilin d modulates mitochondrial f0f1-atp synthase by interacting with the lateral stalk of the complex. J. Biol. Chem..

[81] Porter G.A., Beutner G. (2018). Cyclophilin d, somehow a master regulator of mitochondrial function. Biomolecules.

[82] Chinopoulos C., Adam-Vizi V. (2012). Modulation of the mitochondrial permeability transition by cyclophilin D: Moving closer to f(0)–f(1) ATP synthase?. Mitochondrion.

[83] Bonora M., Bononi A., De Marchi E., Giorgi C., Lebiedzinska M., Marchi S., Patergnani S., Rimessi A., Suski J.M., Wojtala A. (2013). Role of the c subunit of the fo atp synthase in mitochondrial permeability transition. Cell Cycle.

[84] Neginskaya M.A., Solesio M.E., Berezhnaya E.V., Amodeo G.F., Mnatsakanyan N., Jonas E.A., Pavlov E.V. (2019). Atp synthase c-subunit-deficient mitochondria have a small cyclosporine a-sensitive channel, but lack the permeability transition pore. Cell Rep..

[85] Krestinin R., Baburina Y., Odinokova I., Kruglov A., Fadeeva I., Zvyagina A., Sotnikova L., Krestinina O. (2020). Isoproterenol-induced permeability transition pore-related dysfunction of heart mitochondria is attenuated by astaxanthin. Biomedicines.

[86] Feng W., Li W. (2010). The study of ISO induced heart failure rat model. Exp. Mol. Pathol..

[87] Guo R., Zong S., Wu M., Gu J., Yang M. (2017). Architecture of Human Mitochondrial Respiratory Megacomplex I2III2IV2. Cell.

[88] Iwata S., Lee J.W., Okada K., Lee J.K., Iwata M., Rasmussen B., Link T.A., Ramaswamy S., Jap B.K. (1998). Complete Structure of the 11-Subunit Bovine Mitochondrial Cytochrome bc1 Complex. Science.

[89] Zhao R.-Z., Jiang S., Zhang L., Yu Z.-B. (2019). Mitochondrial electron transport chain, ROS generation and uncoupling (Review). Int. J. Mol. Med..

[90] Schlame M., Rua D., Greenberg M.L. (2000). The biosynthesis and functional role of cardiolipin. Prog. Lipid Res..

[91] Chicco A.J., Sparagna G.C. (2007). Role of cardiolipin alterations in mitochondrial dysfunction and disease. Am. J. Physiol. Cell Physiol..

[92] Dolinsky V.W., Cole L.K., Sparagna G.C., Hatch G.M. (2016). Cardiac mitochondrial energy metabolism in heart failure: Role of cardiolipin and sirtuins. Biochim. Biophys. Acta.

[93] Fiedorczuk K., Letts J.A., Degliesposti G., Kaszuba K., Skehel M., Sazanov L.A. (2016). Atomic structure of the entire mammalian mitochondrial complex i. Nature.

[94] Palsdottir H., Lojero C.G., Trumpower B.L., Hunte C. (2003). Structure of the yeast cytochrome bc1 complex with a hydroxyquinone anion qo site inhibitor bound. J. Biol. Chem..

[95] Shinzawa-Itoh K., Aoyama H., Muramoto K., Terada H., Kurauchi T., Tadehara Y., Yamasaki A., Sugimura T., Kurono S., Tsujimoto K. (2007). Structures and physiological roles of 13 integral lipids of bovine heart cytochrome c oxidase. EMBO J..

[96] Dudek J., Cheng I.F., Chowdhury A., Wozny K., Balleininger M., Reinhold R., Grunau S.D., Callegari S., Toischer K., Wanders R.J. (2016). Cardiac-specific succinate dehydrogenase deficiency in Barth syndrome. EMBO Mol. Med..

[97] Dudek J. (2017). Role of Cardiolipin in Mitochondrial Signaling Pathways. Front. Cell Dev. Biol..

[98] Rodrigo R., Libuy M., Feliu F., Hasson D. (2013). Oxidative stress-related biomarkers in essential hypertension and ischemia-reperfusion myocardial damage. Dis. Mark..

[99] Long Q., Yang K., Yang Q. (2015). Regulation of mitochondrial ATP synthase in cardiac pathophysiology. Am. J. Cardiovasc. Dis..

[100] Sinatra S.T. (2009). Metabolic cardiology: An integrative strategy in the treatment of congestive heart failure. Altern. Ther. Health Med..

[101] Chen C., Ko Y., Delannoy M., Ludtke S.J., Chiu W., Pedersen P.L. (2004). Mitochondrial atp synthasome: Three-dimensional structure by electron microscopy of the atp synthase in complex formation with carriers for pi and adp/atp. J. Biol. Chem..

[102] Chen C., Saxena A.K., Simcoke W.N., Garboczi D.N., Pedersen P.L., Ko Y.H. (2006). Mitochondrial atp synthase. Crystal structure of the catalytic f1 unit in a vanadate-induced transition-like state and implications for mechanism. J. Biol. Chem..

